# Shading of the mother plant during seed development promotes subsequent seed germination in soybean

**DOI:** 10.1093/jxb/erz553

**Published:** 2020-01-11

**Authors:** Feng Chen, Wenguan Zhou, Han Yin, Xiaofeng Luo, Wei Chen, Xin Liu, Xingcai Wang, Yongjie Meng, Lingyang Feng, Yuanyuan Qin, Cuiying Zhang, Feng Yang, Taiwen Yong, Xiaochun Wang, Jiang Liu, Junbo Du, Weiguo Liu, Wenyu Yang, Kai Shu

**Affiliations:** 1 School of Ecology and Environment, Northwestern Polytechnical University, Xi’an, China; 2 Key Laboratory of Crop Ecophysiology and Farming System in Southwest China, Institute of Ecological Agriculture, Sichuan Agricultural University, Chengdu, China; 3 College of Agronomy, Shandong Agricultural University, Taian, China; 4 Wuhan Metware Biotechnology Co., Ltd., Wuhan, China; 5 Meteorological Bureau, Heze, Shandong, China; 6 Royal Holloway, University of London, UK

**Keywords:** *Glycine max*, parental environment, phytohormone, pro-anthocyanidins, seed germination, shade, soybean

## Abstract

The effect of shading during seed development on subsequent germination remains largely unknown. In this study, two soybean (*Glycine max*) seed production systems, monocropping (MC) and maize–soybean intercropping (IC), were employed to examine the effects of shading of the mother plant on subsequent seed germination. Compared to the MC soybean seeds, which received light, the developing IC seeds were exposed to shade resulting from the taller neighboring maize plants. The IC seeds germinated faster than the MC seeds, although there was no significant difference in the thickness of the seed coat. The concentration of soluble pro-anthocyanidin in the IC seed coat was significantly lower than that in the MC seed coat. Changes in the concentrations of several types of fatty acids in IC seeds were also observed, the nature of which were consistent with the effect on germination. The expression levels of genes involved in abscisic acid (ABA) biosynthesis were down-regulated in IC seeds, while the transcription levels of the genes related to gibberellin (GA) biosynthesis were up-regulated. This was consistently reflected in decreased ABA concentrations and increased active GA_4_ concentrations in IC seeds, resulting in an increased GA_4_/ABA ratio. Our results thus indicated that shading of the mother plant during seed development in soybean promoted subsequent germination by mediating the biosynthesis of pro-anthocyanidins, fatty acids, and phytohormones.

## Teaser

Shading of the soybean mother plant during seed development promotes subsequent germination by mediating the biosynthesis of pro-anthocyanidins, fatty acids, and the phytohormones ABA and GA.

## Introduction

Seed germination is one of the most important stages of the life-cycle of an angiosperm. In agricultural production systems, the timely and uniform processes of germination and seedling emergence are key determinants of crop yield (Ashraf and Foolad, 2005; Chauhan and Opeña, 2012). Consequently, it is essential to understand the molecular mechanisms that regulate seed germination, and this field of research has attracted the attention of investigators from various plant disciplines, including ecologists, geneticists, physiologists, molecular biologists, and plant breeders.

Both endogenous signals and exogenous environmental cues precisely regulate seed germination, and the underlying molecular mechanisms have been studied both extensively and intensively (Bentsink and Koornneef, 2008; Graeber *et al.*, 2012; Shu *et al.*, 2013, 2016*b*). The phytohormones abscisic acid (ABA) and gibberellins (GAs) have been found to be the most important factors, and the stimulatory effect of GAs and the inhibitory effect of ABA on seed germination are well documented (Finkelstein *et al.*, 2008; Shu *et al.*, 2013, 2016*b*, 2018; Vaistij *et al.*, 2013). Enhanced GA signaling or elevated GA concentrations can accelerate seed germination, resulting from the effect of increased secretion of hydrolytic enzymes on a weakened testa structure (Seo *et al.*, 2006; Holdsworth *et al.*, 2008). In contrast, ABA delays germination, so that seeds of various mutants exhibiting altered ABA biosynthesis or signaling, such as *abi3*, *abi4*, and *abi5*, show changes in germinability compared to the wild-type (Piskurewicz *et al.*, 2008; Kanai *et al.*, 2010; Frey *et al.*, 2012; Shu *et al.*, 2013). ABA acts through the PYR/PYL/RCAR-PP2C-SnRKs signaling pathway (Cutler *et al.*, 2010), and key factors in this pathway regulate germination (Née *et al.*, 2017; Nishimura *et al.*, 2018).

Seed germination is defined by the emergence of the radicle (Bewley, 1997; Finch-Savage and Leubner-Metzger, 2006), which is driven by the energy stored in the seed itself (Eastmond, 2004, 2006; Chen *et al.*, 2016). During the imbibition of oil-containing seeds, the hydrolysis of triacylglycerol releases glycerol and fatty acids, and the conversion of the latter to sugars then fuels germination (Eastmond, 2004, 2006; Quettier and Eastmond, 2009; Theodoulou and Eastmond, 2012). The association between the seed fatty acid concentration and germination ability has been examined. In soybean (*Glycine max*), a negative correlation between the oleic acid concentration and germination vigor has been reported (Bachleda *et al.*, 2017), while in sweet pepper (*Capsicum annuum*), the linoleic acid concentration is positively correlated with germination vigor, with higher concentrations of palmitoleic acid leading to poorer seed germination (Kaymak, 2014). Changes in the concentrations of several fatty acids and sugars during soybean seed germination have also been documented (Zhou *et al.*, 2019).

In addition to the roles of phytohormones and fatty acids described above, polyphenolic compounds such as anthocyanidins and pro-anthocyanidins (PAs) are also involved in the control of seed germination (Jia *et al.*, 2012*b*; Smýkal *et al.*, 2014; MacGregor *et al.*, 2015; Shah *et al.*, 2018; Zhao *et al.*, 2019). These compounds play an indirect restrictive role during seed imbibition by hampering radicle protrusion, and are therefore important determinants of seed coat-regulated germination (Debeaujon *et al.*, 2000). Arabidopsis *TRANSPARENT TESTA 12* (*TT12*) encodes a protein with similarity to prokaryotic and eukaryotic multi-drug secondary transporters, and *tt12* mutant seeds show reduced dormancy and significantly lower PA concentrations than the wild-type (Debeaujon *et al.*, 2001). The *tt12* mutation systematically leads to the complete disappearance of PAs from the seed coat, confirming the negative relationship between PA concentration and germination. In Chinese tallow tree, *Sapium sebiferum*, high concentrations of PAs in the seed coat inhibits the germination processes, during which PAs interact with the metabolic pathways of ABA, GA, and reactive oxygen species (Shah *et al.*, 2018, Preprint). In Arabidopsis, PAs cause a delay in seed germination, and the underlying mechanism has been identified as the maintenance by PAs of high concentrations of ABA (Jia *et al.*, 2012*a*). Further insights have come from studies of sheepgrass (*Leymus chinensis*), in which the LcbHLH92 transcription factor acts as a negative regulator of anthocyanin and PA biosynthesis, and the overexpression of *LcbHLH92* in Arabidopsis results in lower concentrations of PAs and higher germination ability (Zhao *et al.*, 2019).

Most studies on seed germination have focused on the roles of endogenous or environmental cues specifically during imbibition (Kanai *et al.*, 2010; Martínez-Andújar *et al.*, 2011; Shu *et al.*, 2013, 2016*b*; Barrero *et al.*, 2014; Nonogaki *et al.*, 2014; Née *et al.*, 2017), and there have been relatively few studies into the effects of exposure of the parent plant to environmental cues on the germination of subsequent seeds (Kvaalen and Johnsen, 2008; Postma and Ågren, 2015). Further examination of the effects of the parental environmental on subsequent seed germination is therefore required.

Light regulates numerous physiological processes throughout the plant life-cycle. Red light promotes seed germination through repression of the transcription of ABA biosynthesis genes, while far-red light delays germination by inducing the expression of ABA biosynthesis genes (Barrero *et al.*, 2014). The shade environment that results from canopy mixing among neighboring plants, especially under close planting or maize–soybean relay-intercropping systems (Yang *et al.*, 2014, 2015; Liu *et al.*, 2017*b*), markedly affects plant architecture, seed quantity, and quality (Casal, 2013; Yang *et al.*, 2014, 2018; Huang *et al.*, 2018), and is the result of a decreased red:far-red ratio and lower photosynthetic photon flux density. Under shade conditions, plants show a shade-avoidance syndrome, including excessive elongation of the hypocotyl, stem, and petiole, and early flowering (Casal, 2013), which enables them to grow and compete effectively with their neighbors (Casal, 2013; Huang *et al.*, 2018). However, the effects of a parental shade environment during the reproductive stage on the germination of subsequent seeds is largely unknown.

Some previous studies have focused on the parental effects of native ecosystems or species on subsequent plant growth stages, including seed germination (Galloway, 2001; Kvaalen and Johnsen, 2008; Awan *et al.*, 2018). The effects of shading that result from cultivation systems are less well studied and are worthy of examination. Our present study reported that shade during the seed development stage in soybean promotes subsequent germination by regulating the endogenous levels of PAs, fatty acids, and the phytohormones ABA and GAs. We studied seeds produced in two soybean production systems, namely monocropping (MC) and maize–soybean intercropping (IC). The control group seeds (MC) received light, while the IC seeds suffered from shade stress during seed development from the taller neighboring maize plants. The results showed that IC seeds germinated faster than MC seeds. Subsequent biochemical analysis revealed that the concentrations of soluble PAs and several fatty acids in the IC seeds were altered, and these were consistent with the changes observed in germination. qPCR assays showed that the transcription levels of ABA biosynthesis genes were down-regulated in IC seeds, while the transcription levels of GA biosynthesis genes were up-regulated. Decreases in ABA and increases in active GA_4_ concentrations were consistently detected in IC seeds, so that the GA_4_/ABA ratio was increased. Our results improve our understanding of the effects of shading of the parent plant during seed development on the germination of the subsequent seeds.

## Materials and methods

### Seed production

Using a maize–soybean (*Zea mays*–*Glycine max*) relay intercropping system previously developed by our group (Yang *et al.*, 2014, 2015; Liu *et al.*, 2017*a*), we obtained intercropped soybean seeds (IC) and monocropped seeds (MC). The seeds used in this study were produced and studied at two different locations in 2016–2017, namely Heze in Shandong Province, China (35°15′N, 115°25′E) and Chengdu in Sichuan Province (30°33′N, 103°38′E). Climatic data for these locations during the soybean growing seasons in 2016 to 2017 are shown in Supplementary Table S1 at *JXB* online. The MC seeds that were subject to light during development acted as the control, while the IC seeds suffered shade stress resulting from the canopy of the taller neighboring maize plants during development. At the Shandong site, the soybean cultivar Qihuang 34 (QH-34) and the maize cultivar Jundan 20 (JD-20) were used, whilst at Sichuan, the soybean cultivars Nandou 12 (ND-12) and C-103, and the maize cultivar Zhenghong 505 (ZH-505) were used.

The maize–soybean intercropping and soybean monocropping patterns were established according to our previous protocol (Yang *et al.*, 2014, 2015; Liu *et al.*, 2017*a*). In detail, in the relay intercropping system there were two rows of maize alternated with two rows of soybean, with a row spacing of 0.4 m and a distance between plants within a row of 0.6 m. For the soybean monocropping, the row spacing was 0.5 m. The area of each experimental plot was 36 m^2^ (6×6 m). At noon at the Shandong site, the red to far-red (R/FR) ratio at the top of the soybean canopy in the intercrop was ~0.4 and the photosynthetic photon flux density (PPFD) was ~740 µmol m^−2^ s^−1^ (49% of that of the monoculture soybean plants). For the Sichuan site, the R/FR ratio was ~0.65 and the PPFD was ~1050 µmol m^−2^ s^−1^ (62% of that of the monoculture soybean plants). The length of the photoperiods and the day/night ratios during the growing seasons for the two sites are given in Supplementary Table S2. All the experiments consisted of a randomized complete block design with three replicates. In addition, we also grew soybean plants of the ND-12 variety in a greenhouse under artificial shade produced by green filters, as described previously (Sasidharan *et al.*, 2008). The harvested seeds were selected for subsequent experiments as described previously (Shu *et al.*, 2017; Zhou *et al.*, 2019).

### Seed germination

Samples of 20 soybean seeds were placed on two layers of medium-speed qualitative filter papers in Petri Dishes (diameter 9 cm) to which 15 ml water was added, and they were then incubated at 25 °C in the dark in an incubator (Versatile Environmental Test Chamber MLR-350H, Sanyo). The time-course of germination was then monitored. Four replicates were used. Seed germination was defined as the emergence of the radicle. At 48 h after sowing, the radicles were detached using a knife, and the root length, fresh weight, and dry weight of each 20-seed sample were measured. The ImageJ software was used to determine the radicle length.

A detailed microscopic examination of the seeds was made during germination *sensu stricto*. Images of seeds were taken under a M165C stereomicroscope (Leica, 10× magnification) between 0–18 h indicated after sowing in the Petri dishes, with particular focus on the radicle.

### Extraction and quantification of fatty acids

Various types of fatty acids were extracted from the soybean seeds at different stages of development according to our previously published protocol (Liu *et al.*, 2016; Zhou *et al.*, 2019). The development stages were characterized according to Fehr and Caviness (1977) and Han *et al.* (2006). Seeds were ground in liquid nitrogen and then freeze-dried. Powdered samples of 50 mg were placed in 10-ml centrifuge tubes and 3 ml of n-hexane was added. The samples were extracted ultrasonically (at 40 kHz) for 15 min and kept at room temperature for 3 h. The solution was centrifuged for 15 min at 13 200 *g* at 4 °C. Then, 3 ml of 0.4 M methanolic potassium hydroxide solution was added to the supernatant, the tubes were subjected to vortex oscillation for 30 s, and then kept at room temperature for 1 h. The upper liquid layer was transferred to a 5-ml capacity bottle, made up to full volume by addition of n-hexane, and then injected into a GCMS-QP2010 system (Shimadzu) through a 0.45-μm organic-phase filter.

The fatty acid concentrations of the samples were quantified by comparing the retention times and area spectra with a fatty acid methyl ester (FAME) standard mixture containing 37 compounds (Nu-Chek-Prep Inc., USA). This standard mixture included oleic, linoleic, palmitic, stearic, and α-linolenic acid. Three biological replications were performed. To determine the relationships among the levels of fatty acids in the different samples, heat maps were created using the Adobe Illustrator software as described previously (Liu *et al.*, 2016; Zhou *et al.*, 2019).

### Measurement of sugars

Developing soybean seeds at different stages were sampled, heated at 105 °C for 30 min, and then dried at 80 °C until constant weight was obtained. The dried samples were ground, and 100 mg samples of powder were placed in 10-ml centrifuge tubes to which 4 ml of 80% (v/v) ethanol was then added. The tubes were placed in a water bath at 80 °C for 40 min and then centrifuged at 4500 *g* for 10 min. This extraction procedure was repeated three times, and the combined supernatants were then diluted to 50 ml with ethanol (80%). The contents of sucrose, fructose, total soluble sugars, and reducing sugars were then quantified as described previously (Cai *et al.*, 2016; Shi *et al.*, 2016; Zhou *et al.*, 2019).

### Measurement of thickness of seed coat

The thickness of the soybean seed coat was measured using a M165C stereomicroscope (Leica). At least 10 seeds per sample were used, and the seed coats were peeled off and each one was measured five times at different angles.

### Measurement of pro-anthocyanidins in the seed coat

Soluble and insoluble PAs were extracted from the seed coats according to the protocol described by Senda *et al.* (2017). The seed coats were peeled off, ground in liquid nitrogen, and freeze-dried. Samples of 50 mg of the powder were extracted in the dark at 4 °C for 1 h in 1 ml 70% (v/v) acetone aqueous solution containing 0.1% (w/v) ascorbic acid. The samples were centrifuged at 11 000 *g* for 10 min, and the extraction procedure was repeated three times (2×1 h, and once overnight). The residue was then used to quantify the level of insoluble PAs, whilst the combined supernatants were made up to 4 ml with the acetone aqueous solution. The supernatant was mixed with 3 ml of diethyl ether at –20 °C, and then the lower phase was transferred to another centrifuge tube for determination of soluble PAs. The contents of soluble and insoluble PAs were determined using the method described previously by Senda *et al.* (2017).

### Gene expression analysis

Quantitative PCR (qPCR) was performed as described previously (Shu *et al.*, 2013; Zhou *et al.*, 2019). Total RNA was extracted from dry and imbibed soybean seeds (0–9 h after sowing) Then, 2-μg samples were treated with DNase I and reversed-transcribed using Moloney Murine Leukemia Virus Reverse Transcriptase (200 units per reaction; Promega). qPCR was performed using a QuantStudio 6 Flex Real-Time PCR System (ThermoFisher Scientific) and Vazyme™ AceQ qPCR SYBR Green Master mix. The expression levels of genes involved in ABA/GA biosynthesis and signaling cascades were analysed. Gene expression was quantified at the logarithmic phase using the expression of the housekeeping *GmTubulin* gene as an internal control. Expression analysis of each gene was repeated three times. Primers sequence are given in Supplementary Table S3.

### Quantification of ABA and GA in seeds

Quantification of ABA and GA were performed according to the methods described in our previous studies (Chen *et al.*, 2011; Shu *et al.*, 2013, 2016*a*).

For ABA, dry and imbibed (6 h after sowing, 400 mg) soybean seeds were ground in liquid nitrogen and extracted for 24 h in methanol containing D6-ABA (OIChemIm Co. Ltd.) as an internal standard. Purification was performed using an Oasis Max solid-phase extract cartridge (Waters) and eluted with 5% formic acid in methanol. The elution was then dried and reconstituted, and injected into a LC–tandem MS system consisting of an Acquity ultra-performance LC (Acquity UPLC; Waters) and a triple-quadrupole tandem MS (Quattro Premier XE; Waters). Three biological replications were performed.

For GA, dry and imbibed (6 h after sowing, 400 mg) soybean seeds were ground in liquid nitrogen and extracted with 80% (v/v) methanol. GA d2 isotope standards were added to the samples before grinding. The crude extracts were purified by reversed-phase solid-phase extraction, ethyl-ether extraction, and derivatization. The resulting mixture was injected into a capillary electrophoresis-MS system (Agilent Technologies) for quantitative analysis. Three biological replications were performed.

### Statistical analysis

The data were analysed using Student’s *t*-test. Time-to-event analysis of seed germination data was performed using the R function lifetab () in the KMsurv package, according to the method described by McNair *et al.* (2012).

## Results

### Ecological characterization of inter- and monocropping seeds

**Fig. 1.**
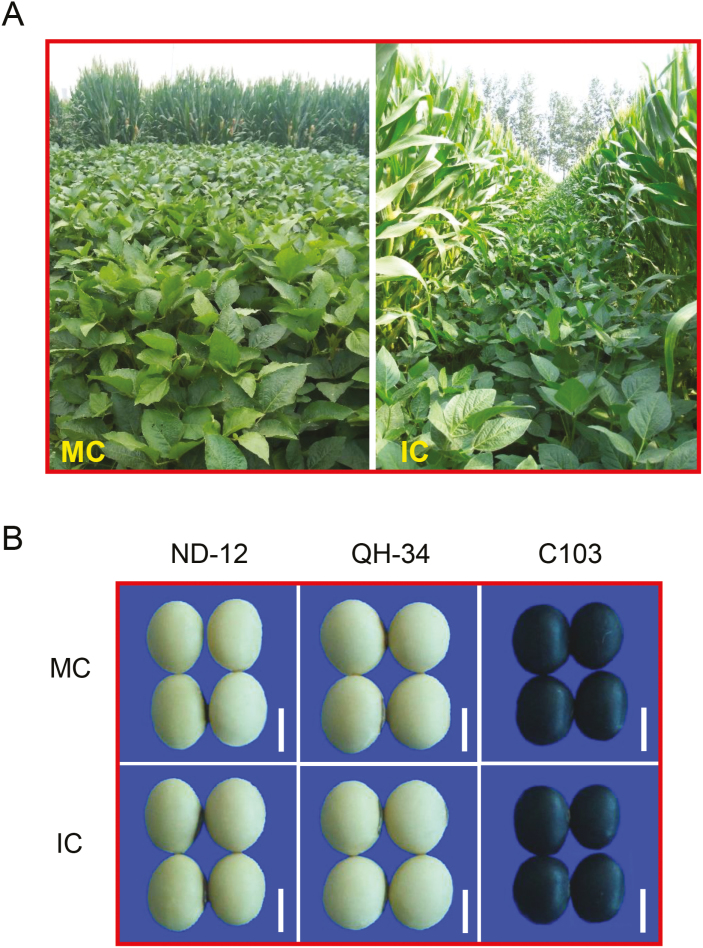
Monocropping and intercropping of soybean in the field, and morphology of seeds. (A) Representative images of monocropping (MC) and maize–soybean intercropping (IC) using the cultivar QH-34. (B) Representative images of seeds harvested from MC and IC plants. The cultivars are indicated at the top. Scale bars are 5 mm. (This figure is available in colour at *JXB* online.)

To examine the effects of the shade environment of the parental plant on the germination of subsequent seeds, soybean plants were grown under two different production systems, namely monocropping (MC, control treatment) and intercropping (IC) with maize. As already demonstrated by our previous studies (Yang *et al.*, 2014, 2015; Liu *et al.*, 2017*a*), the IC soybean plant canopy suffered from shade stress imposed by the taller neighboring maize plants during seed development, in contrast to the MC plants that received the light (Fig. 1A). The R/FR ratio and the PPFD received by the IC soybean plants were decreased significantly compared to the MC plants (Yang *et al.*, 2014, 2015; Liu *et al.*, 2017*a*). There were no obvious morphological differences between the IC and MC soybean seeds (Fig. 1B), which were comparable with regard to their 100-seed weight, length, width, and the ratio of length to width (Supplementary Fig. S1).

### Intercropping seeds germinate faster than monocropping seeds

**Fig. 2.**
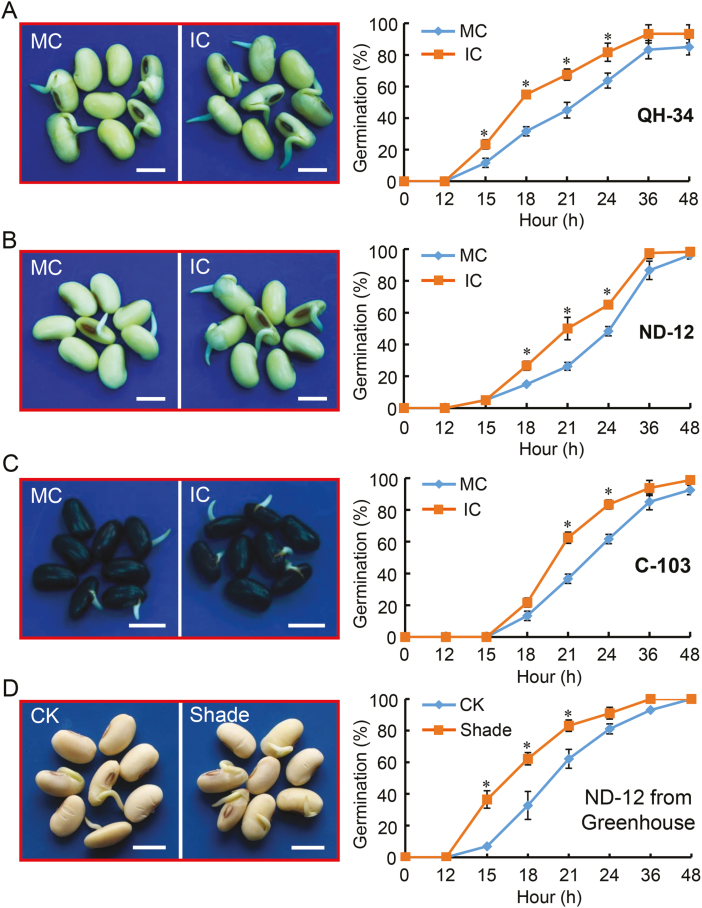
Soybean seeds from parent plants subjected to shading germinate faster than seeds from plants without shading. (A–C) Representative images (left) and germination patterns (right) of seeds from plants grown with monocropping (MC) and with intercropping (IC). (A) Seeds of the QH-34 cultivar produced at the Shandong experimental site; (B) seeds of the ND-12 cultivar produced at the Sichuan experimental site; and (C) seeds of the C-103 cultivar produced at the Sichuan experimental site. The images were taken at 21 h after sowing. (D) Representative images and germination patterns of seeds of ND-12 from plants that were either not shaded (control, CK) or subjected to artificial shade in a greenhouse. The images were taken at 18 h after sowing. All scale bars are 10 mm. All data are means (±SD) of four replicates. Significant differences were determined using Student’s *t*-test: *P<0.05. (This figure is available in colour at *JXB* online.)

**Fig. 3.**
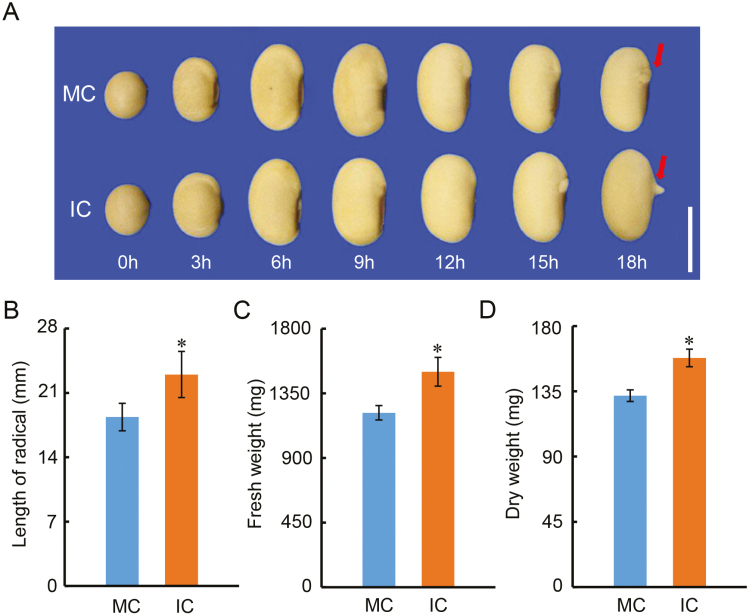
Development of soybean seeds from parent plants subjected to shading is faster than that of seeds from unshaded plants. (A) Representative images of seeds during the course of imbibition of the cultivar ND-12 from plants grown at the Sichuan experimental site with monocropping (MC) and with intercropping (IC). The protrusion of the radicle is indicated by arrows. The scale bar is 10 mm. (B) Length of the radicle, and (C) fresh and (D) dry weights of germinated seeds at 48 h after sowing. Data are means (±SE) of four replicates. Significant differences were determined using Student’s *t*-test: *P<0.05. (This figure is available in colour at *JXB* online.)

Seed germination assays conducted on the different soybean cultivars at the two experimental sites demonstrated that the IC seeds germinated faster than the MC seeds (Figs 2, 3, Supplementary Figs S2, S3).

For the QH-34 cultivar, the seeds of which were produced in Shandong Province, the germinability of IC seeds was clearly higher than that of MC seeds (Fig. 2A). For example, at 18 h after sowing the percentage germination of IC soybean seeds was nearly 60% whilst that of MC seeds was only 30%. The faster germination of IC seeds was apparent throughout the germination processes. We performed similar assays with seeds from two other cultivars, ND-12 and C103, which come from different genetic backgrounds to QH-34 and which were produced in Sichuan Province ~1400 km from the Shandong site. A faster-germination phenotype of IC seeds was also observed for these two cultivars (Fig. 2B, C). In addition, we also performed time-to-event analysis of the germination data using the KMsurv package and found that the probability of not germinating was consistently higher for MC seeds compared to IC seeds for all three genotypes (Supplementary Fig. S3A–C).

The early visible stages of germination involve several processes, including rupture of the seed coat and protrusion of the radicle. We therefore examined germination under a microscope and found that rupture and radicle protrusion occurred earlier in IC seeds than in MC seeds (Fig. 3A). In contrast to MC seeds, at 15 h after sowing the IC seeds showed obvious rupture of the seed coat, and at 18 h the radicle of IC seeds was markedly longer than that of MC seeds.

We next examined the effects of maternal plant shade on post-germination seedling growth, using seeds of the ND-12 cultivar produced at the Sichuan Province site. The length of the radicle of germinated IC seeds was significantly longer than that of MC seeds (Fig. 3B), and there were also significant increases in the fresh and dry weights of the roots of the IC seeds compared to the MC seeds (Fig. 3C, D). Seeds of the QH-34 cultivar produced at the Shandong site were also examined and their germination characteristics showed trends similar to those of ND-12 (Supplementary Fig. S2).

In order to further confirm the seed germination phenotype of IC seeds caused by the maternal plant shade environment, we produced seeds from plants grown in a greenhouse in which shade was imposed artificially by the use of green filters. The results of the germination assays were consistent with those of seeds obtained under natural shading in the field (Fig. 2D), and the results of time-to-event analysis were also consistent (Fig. S3D). Taken together, these phenotypic analyses consistently demonstrated that maternal shading increased the germination of subsequent seeds. Since the differences in germination between IC and MC seeds were consistent across the three cultivars produced at the different locations and between natural and greenhouse conditions (Figs 2, 3, Supplementary Figs S2, S3), we selected one cultivar, ND-12, for further biochemical and gene transcription analysis.

### Variations in fatty acid and sugar concentrations between inter- and monocropping seeds

Several previous studies have shown that during seed development in oilseed crops (such as soybean, rapeseed, and Arabidopsis), the sucrose produced by photosynthesis is converted to hexoses for the biosynthesis of oil (triacylglycerol, TAG), while fatty acids are important mediators that are necessary for subsequent germination and early seedling establishment (Eastmond, 2004, 2006; Quettier and Eastmond, 2009; Theodoulou and Eastmond, 2012; Xu and Shanklin, 2016). In order to determine the mechanisms underlying the faster germination phenotype of the IC seeds, we therefore examined the concentrations of several sugars and fatty acids between IC and MC seeds during their development in the ND-12 cultivar.

Examination of sugars suggested that there were no significant differences between the IC and MC seeds at most of the sampled time points, from stages R5 (seed length ~3 mm) to R8 (fully mature seed; Supplementary Fig. S4).

**Fig. 4.**
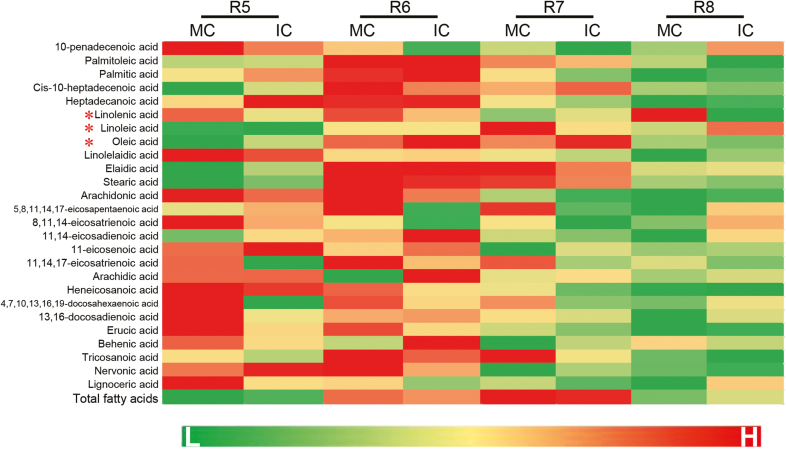
Variation of contents of fatty acids during development of soybean seeds from parent plants grown under different shading environments. Data are from the cultivar ND-12 from plants grown at the Sichuan experimental site with monocropping (MC) and with intercropping (IC). The developmental stages are: R5, seed length ~3 mm; R6, green seed fills the pod cavity; R7, one normal pod reaches mature pod color; and R8, fully mature seed. A heatmap of fatty acid content is shown and the scale indicates the variation from low (L) to high (H) content. Fatty acids that are known to be associated with the control of seed germination are indicated (*). (This figure is available in colour at *JXB* online.)

For fatty acids, GC-MS analysis revealed that, by the R6 stage of development (green seed fills the pod cavity), most of the concentrations had peaked in both the IC and MC of seeds (Fig. 4). From the R6 to the R8 stage, the concentrations decreased in both seed types but whilst it was gradual in the MC seeds, the decline was much steeper in IC seeds, especially at stage R7 (one normal pod reaches mature pod color). At stage R8, differences were observed between the IC and MC seeds in the concentrations of some fatty acids that are known to be involved in seed germination, namely oleic, linolenic, and linoleic acid, and this was consistent with the faster germination phenotype of the IC seeds. Thus, the concentrations of oleic and linolenic acid decreased in IC seeds compared to MC seeds, while the concentration of linoleic acid increased (Fig. 4). Overall, these results indicated that the shade environment of the parental plant influenced the concentrations of fatty acids during seed development, some of which are known to be associated with germination processes.

### Variations in pro-anthocyanidin concentrations in inter- and mono-cropping seed coats

**Fig. 5.**
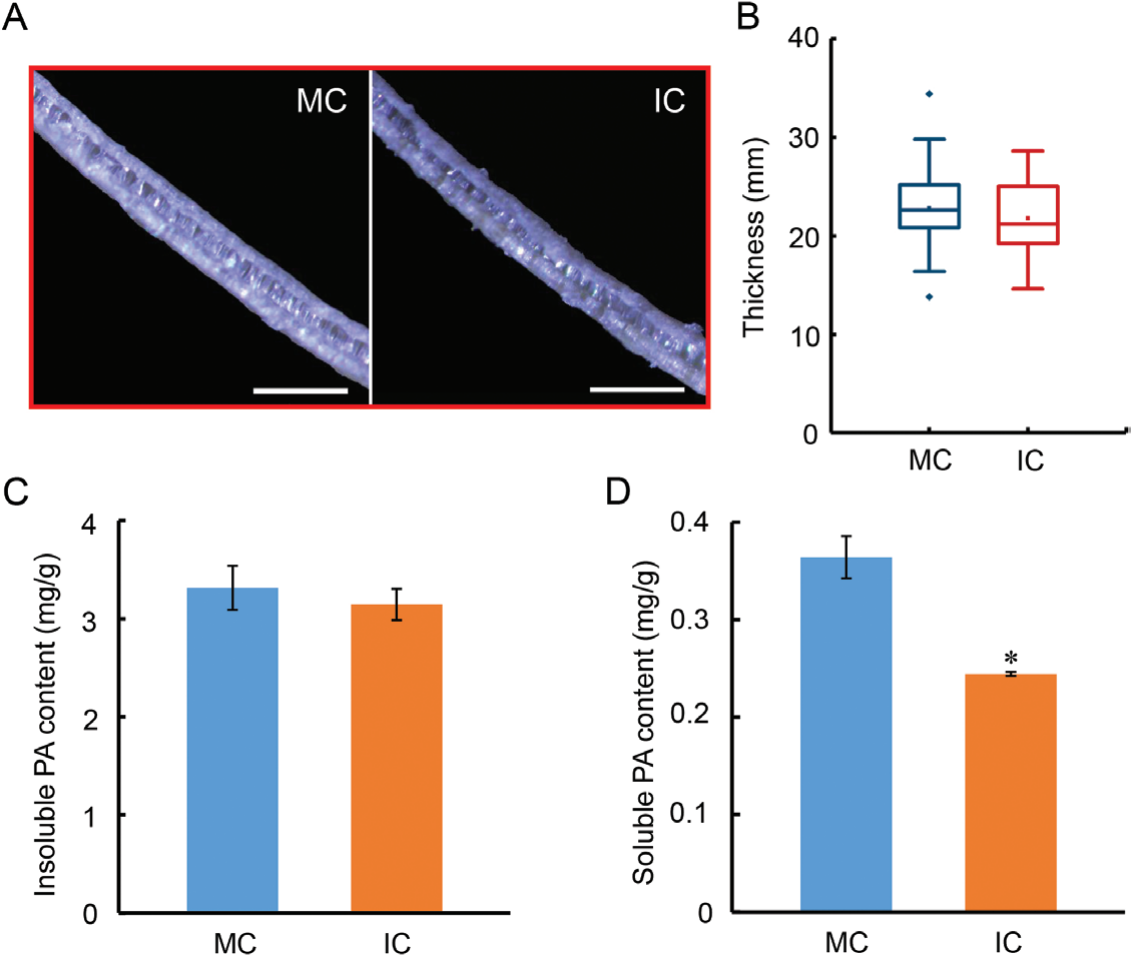
Variation of the thickness of the seed coat and its content of pro-anthocyanidins (PAs) in soybean seeds from parent plants grown under different shading environments. Data are from the cultivar ND-12 from plants grown at the Sichuan experimental site with monocropping (MC) and with intercropping (IC). (A) Representative images of seed coats from MC and IC seeds. The scale bars are 50 μm. (B) Boxplots of the seed coat thickness. At least 10 seeds were used to measure the thickness, and each seed coat was measured five times at different angles. Contents of (C) insoluble and (D) soluble PAs in the seed coats. Data are means (±SE) of four replicates. Significant differences were determined using Student’s *t*-test: *P<0.05. (This figure is available in colour at *JXB* online.)

Previous studies have demonstrated that the thickness and composition of the seed coat play important roles during germination (Noodén *et al.*, 1985; Smýkal *et al.*, 2014; MacGregor *et al.*, 2015; Shah *et al.*, 2018, Preprint). We therefore examined the thickness of the seed coats of the MC and IC seeds of the ND-12 cultivar, but found that there was no significant difference between the two (Fig. 5A, B). We then examined the concentrations of pro-anthocyanidins (PAs) in the seed coats. Whilst the concentrations of insoluble PAs were not significantly different (Fig. 5C), the concentrations of soluble PAs in the IC seed coat were significantly lower than those in the MC seeds (Fig. 5D). According to previous studies, the Flowering Locus T (FT) protein inhibits the biosynthesis of PAs (Chen *et al.*, 2014), and in soybean *GmFT2a* is a key regulator that mediates reproductive development (Liu *et al.*, 2018). We therefore determined the expression level of *GmFT2a*, and found that its transcription in IC seeds was higher than that in MC seeds (Supplementary Fig. S6), which was consistent with the decrease in concentration of PAs (Fig. 5D). To further test the robustness of these findings, we also examined the QH-34 cultivar and found that the results were similar to those observed for ND-12 (Supplementary Fig. S5). Taken together, our results indicated that the shade environment of the parent plant had no effect on the thickness of the seed coat or its concentration of insoluble PAs, but shading did negatively affect the concentration of soluble PAs.

### Parental shading increases GA biosynthesis but decreases ABA biosynthesis

**Fig. 6.**
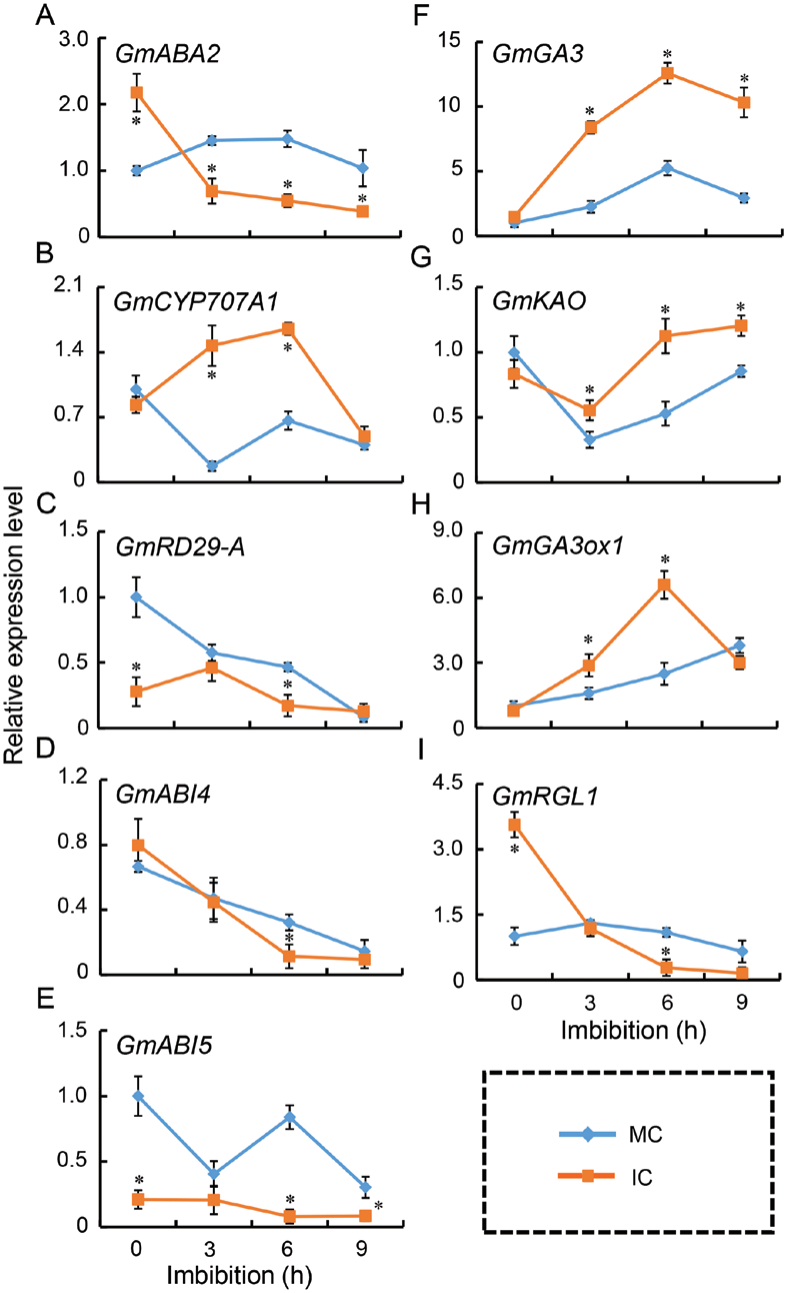
Gene expression during imbibition of soybean seeds from parent plants grown under different shading environments. Data are from the cultivar ND-12 from plants grown at the Sichuan experimental site with monocropping (MC) and with intercropping (IC). Gene expression was determined using qPCR assays during the course of the imbibition process with the housekeeping gene *GmTubulin* used as the internal control. (A) ABA biosynthesis gene *GmABA2*; (B) ABA inactivation gene *GmCYP707A1*; (C–E) positive regulator genes of ABA signaling (C) *GmRD29A*, (D) *GmABI4*, and (E) *GmABI5*; (F–H) GA biosynthesis genes (F) *GmGA3*, (G) *GmKAO*, and (H) *GmGA3ox1*; and (I) negative regulator gene of GA signaling *GmRGL1*. Data are means ±SD of four replicates. Significant differences were determined using Student’s *t*-test: *P<0.05. (This figure is available in colour at *JXB* online.)

Given that the phytohormones ABA and GAs play key roles in seed germination processes (Bewley, 1997; Shu *et al.*, 2013, 2016*b*; Nishimura *et al.*, 2018), our next step was to compare the transcription patterns of key genes involved in their biosynthesis/signaling pathways during germination *sensu stricto*. The results showed that the expression of the ABA biosynthesis gene *GmABA2* was down-regulated in IC seeds during imbibition (Fig. 6A), while the transcription of the ABA inactivation gene *GmCYP707A1* increased (Fig. 6B). The expression levels in IC seeds of *GmRD29-A*, *GmABI4* and *GmABI5*, which encode positive regulators of the ABA signaling pathway, were significantly lower than those in MC seeds at most of the sampled time-points (Fig. 6C–E). qPCR assays showed that the expression levels of the GA biosynthesis genes *GmGA3ox1*, *GmKAO*, and *GmGA3* were significantly higher during seed imbibition in IC seeds than in MC seeds (Fig. 6FH), while the expression of *GmRGL1*, a negative mediator in the GA signaling pathway, was down-regulated during imbibition in IC seeds,compared to MC seeds (Fig. 6I). The results therefore indicated that parental shading negatively regulated ABA biosynthesis and positively mediated GA biosynthesis via transcriptional control of key genes, acting to promote germination in IC seeds significantly more than in MC seeds.

**Fig. 7.**
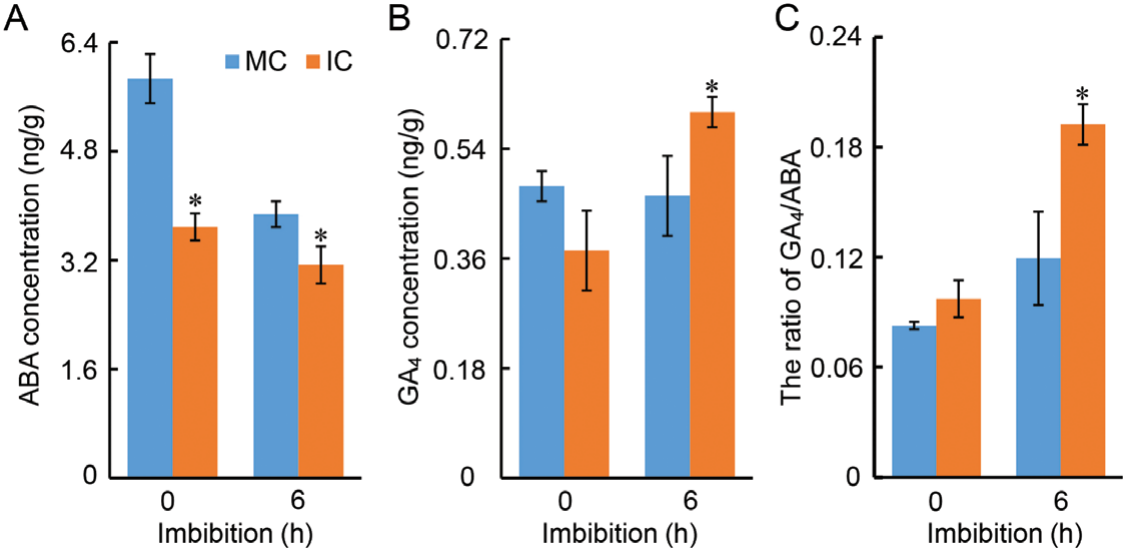
Variation in ABA and GA_4_ concentrations during imbibition of soybean seeds from parent plants grown under different shading environments. Data are from the cultivar ND-12 from plants grown at the Sichuan experimental site with monocropping (MC) and with intercropping (IC). (A) ABA concentration and (B) GA_4_ concentration in dry (0 h) and imbibed seeds (6 h). (C) Ratio of GA_4_ and ABA concentrations. Data are means (±SE) of three replicates. Significant differences between means within a time-point were determined using Student’s *t*-test: *P<0.05. (This figure is available in colour at *JXB* online.)

We next examined ABA and GA_4_ during imbibition and found that the ABA concentration declined in both IC and MC seeds, but it was significantly lower in IC seeds (Fig. 7A). In contrast, the active GA_4_ concentration increased in IC seeds during imbibition whilst remaining unchanged in MC seeds (Fig. 7). As a consequence, the GA_4_/ABA ratio in IC seeds was significantly higher than in MC seeds after 6 h imbibition (Fig. 7C). These results were consistent with the gene expression data (Fig. 6) and the seed germination phenotypes for the two seed types (Figs 2, 3, Supplementary Figs S2, S3).

## Discussion

Our data for seed germination phenotypes, contents of fatty acids and soluble PAs, gene expression, and concentrations of ABA and GAs indicated that parental shading during seed development in soybean promoted the subsequent germination of seeds. We propose that the molecular and physiological mechanisms underlying this positive effect of shade are as follows. Parental shading results in a reduction of biosynthesis of soluble PAs and changes the concentrations of several fatty acids that are associated with seed germination control, namely α-linolenic, oleic, and linoleic acid. In addition, increased biosynthesis of active GA_4_ and decreased biosynthesis of ABA during imbibition of seeds that develop under shade are also responsible for the faster germination. Our study therefore contributes to a better understanding of the effects of environmental cues during the seed development stage on the subsequent processes of germination.

### Parental environmental cues regulate subsequent development stages in offspring

One of the most interesting topics within developmental biology is whether and how parental environment cues have transgenerational influences on development processes, epigenetic modifications, and molecular adaptations of the offspring. There have been numerous studies on this topic in the animal kingdom; for example, adverse or unfavorable prenatal environments can cause metabolic diseases in the offspring (Radford *et al.*, 2014; Vogt *et al.*, 2014; Bauer *et al.*, 2016; Haire-Joshu and Tabak, 2016; Sweeney and MacBeth, 2016). There have also been some pioneering studies that have examined the effects of parental environmental signals on subsequent growth and development in plants.

In Norway spruce (*Picea abies*), the timing of bud set is regulated by the memory of temperature during zygotic and somatic embryogenesis (Kvaalen and Johnsen, 2008), with plants from warmer seed production areas being taller and setting buds later compared with plants from colder parental environments. Parental reproductive temperatures also regulate root growth in the offspring of Arabidopsis, as determined by Blödner *et al.* (2007) who found that seed progeny from a warm parental environment exhibit faster germination, faster root elongation, greater leaf biomass, and increased seed production at various temperatures compared with seed produced in a cold parental environment.

With regard to the control of seed germination, Chen *et al.* (2014) showed that the temperature environment of the parent in Arabidopsis is transduced into a signal through *Flowering Locus T* (*FT*) via the silique phloem, with FT mediating the degree of seed dormancy through inhibition of PA synthesis in the seeds, resulting in a change in the concentration of tannins in the seed coat. Another study in *Brassica oleracea* has shown that exposure to an environmental stress during the reproductive stage has a large impact on seed performance, including germination speed and resistance to controlled deterioration (Awan *et al.*, 2018). Pioneering investigations such as these have suggested that parental environment cues can play key roles in the subsequent growth and development in the progeny.

### Positive effects of parental shading on subsequent seed germination

Most studies of germination have focused on the roles of endogenous or environmental cues specifically during seed imbibition (Kanai *et al.*, 2010; Martínez-Andújar *et al.*, 2011; Shu *et al.*, 2013, 2016*b*; Barrero *et al.*, 2014; Nonogaki *et al.*, 2014; Née *et al.*, 2017). Although some reports have been published on the effects of parental environmental cues on the subsequent germination of the seeds generated (Kvaalen and Johnsen, 2008; He *et al.*, 2014; Postma and Ågren, 2015; Awan *et al.*, 2018), the detailed regulatory mechanisms remain largely unknown, especially the roles of exposure to diverse abiotic stress cues during the development stage on subsequent seed germination. This is in contrast to the more detailed knowledge that is available in the animal kingdom.

**Fig. 8.**
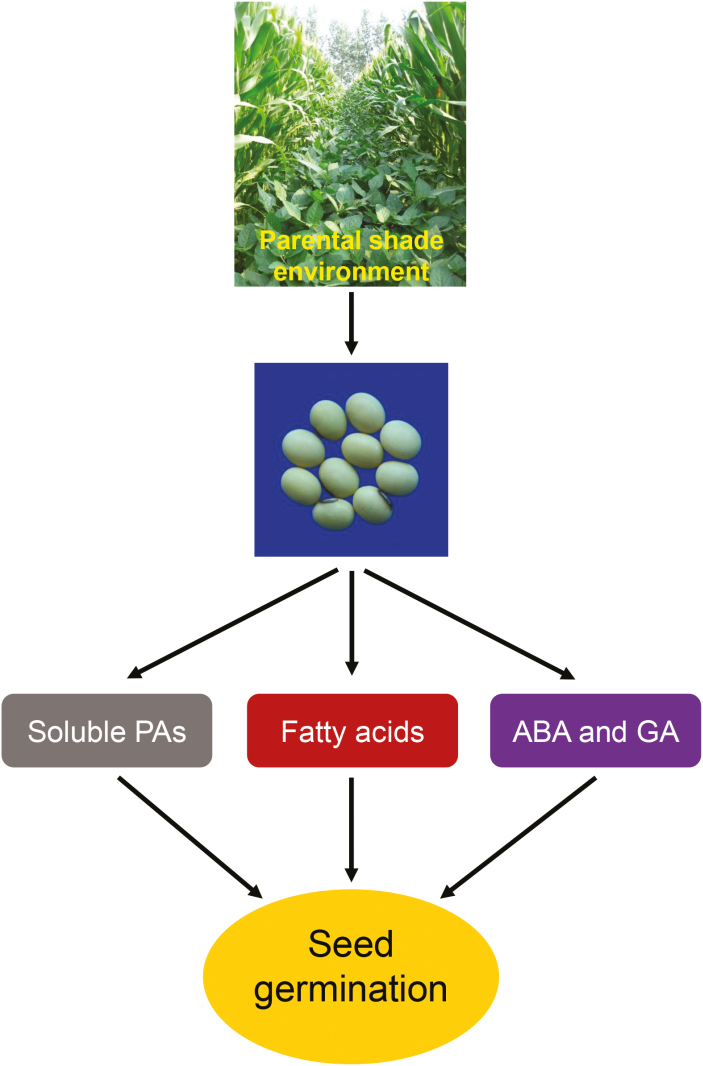
Summary of the effects of shading of the mother plant on the germination of subsequent seeds in soybean. Shading during seed development impairs the biosynthesis of soluble pro-anthocyanidins (PAs) and changes the levels of several fatty acids that are associated with the control of seed germination. In addition, there is increased biosynthesis of active GA_4_ and decreased biosynthesis of ABA during seed imbibition. These effects combine to result in faster germination of seeds that have developed under shade conditions. (This figure is available in colour at *JXB* online.)

There is limited information available on the relationship between shading of the parent plant and the germination characteristics of the seed produced. Red light is known to promote germination while far-red light inhibits the red light-induced processes in some species (Bewley, 1997). Some studies have detected an inhibitory effect of shade on germination (Frankland and Taylorson, 1983; Mathews, 2006; Lee and Lopez-Molina, 2012), but they focused only on the effects of shade during seed imbibition, rather than examining the effects of shade stress of the parent on subsequent seed germination. Early studies indicated that Arabidopsis seeds that had developed under a low ratio of FR to R light germinated faster than seeds grown under a high ratio (McCullough and Shropshire, 1970; Hayes and Klein, 1974). It has been suggested that subsequent seed germination processes are regulated by the spectral quality to which the parent plant was exposed (Chahtane *et al.*, 2017); however, the molecular mechanisms underlying this have remained elusive. In our present study we used seeds from three different soybean cultivars produced under different environmental conditions (at two different locations), as well as seeds produced in greenhouses, and found that the parental shade signal promoted subsequent germination processes (Figs 2, 3, Supplementary Figs S2, S3). Physiological and biochemical analyses showed that parental shading attenuated the biosynthesis of soluble PAs (Fig. 5) and resulted in changes in the concentrations of several fatty acids (Fig. 4), results that were consistent with the patterns of germination. The parental shade signal also down-regulated ABA biosynthesis while up-regulating GA biosynthesis in seeds produced by shaded parent plants, via effects on the transcription of specific genes (Figs 6, 7). Overall, our study demonstrated that shading of the parent during the reproductive stage increased the speed of soybean seed germination via mediation of the biosynthesis of soluble PAs, key fatty acids, and the phytohormones ABA and GA (Fig. 8).

Phytochromes sense R and FR light (Klose *et al.*, 2015; Pham *et al.*, 2018), and phyA and phyB are also involved in the regulation of seed germination through auxin and ABA-mediated pathways in Arabidopsis (Lee *et al.*, 2012; Ibarra *et al.*, 2013). Compared to the extensive studies of the molecular functions of phyA and phyB in model plants, there are have been few studies of GmphyA and GmphyB in soybean. Some studies have focused on the genetic redundancy of soybean phytochromes (Liu *et al.*, 2008; Wu *et al.*, 2013), and have also shown that *GmPHYA*s are involved in post-flowering photoperiod responses (Xu *et al.*, 2013). Given the importance of photoreceptors in plant shade responses (Fraser *et al.*, 2016), we speculate that in soybean, GmphyA and GmphyB might sense the change of ratio of R:FR under shaded environments during seed development and then mediate the subsequent seed germination processes through as yet unknown cascades. This is clearly worthy of future study.

In the field of animal research, it is know that under-nourishment during the prenatal stage alters DNA methylation in the germline of the offspring, compromising the development of male offspring (Radford *et al.*, 2014). Our current work provides a good case study for investigating cross-generational effects in plants induced by environmental cues. In ongoing research, we are examining the underlying genetic mechanisms using epigenetic approaches, including genomic DNA methylation and other molecular effects, and we hope that the precise mechanisms underlying the positive effect of parental shade signals on subsequent seed germination will be uncovered in the near future.

## Supplementary data

Supplementary data are available at *JXB* online.

Fig. S1. Analysis of agronomic traits of MC and IC seeds of the different cultivars.

Fig. S2. Length of radicle, and fresh and dry weights of MC and IC seeds during imbibition for the cultivar QH-34.

Fig. S3. Germination phenotypes of IC and MC seeds of the different cultivars as determined by life-table estimates.

Fig. S4. Effects of parental shading on sugars level during different stages of development of IC and MC seeds in the ND-12 cultivar.

Fig. S5. Seed coat thickness and content of PAs in IC and MC seeds in the cultivar QH-34.

Fig. S6. Expression of *GmFT2a* in MC and IC seeds in the ND-12 cultivar.

Table S1. Air temperature, rainfall, and duration of sunshine during the 2016–2017 soybean growing seasons at the two experimental locations.

Table S2. Data for daylength during the 2016–2017 soybean growing seasons at the two experimental locations.

Table S3. Sequences of primers used in this study.

erz553_suppl_supplementary_tables_S1_S3

erz553_suppl_supplementary_figures_S1_S6

## 

**Author contributions:** KS conceived and designed the study; FC, WZ, HY, X-FL, WC, XL, WW, YM, LF, YQ, FY, TY, X-CaW, X-ChW, JL, JD, and WL performed the experiments; CZ supplied the climatic data for the Shandong location; KS, WY, and FC analysed the data; KS wrote the paper.
