# Supplementary material for: Shading of the mother plant during seed development promotes subsequent seed germination in soybean
Source: J Exp Bot. 2020 Jan 11;71(6):2072–84. doi: 10.1093/jxb/erz553 (PMC7242070; doi:10.1093/jxb/erz553)
Supplement: erz553_suppl_supplementary_tables_S1_S3 [file erz553_suppl_supplementary_tables_s1_s3.pdf]

Supplemental Tables

Supplemental Table S1. Air temperature, rainfall and sunshine duration in both locations (Shandong and Sichuan) during soybean growing seasons from 2016 to 2017.

Year	Average air temperature (°C)					Monthly total rainfall (mm)					Monthly sunshine duration (h)				
	Jun.	Jul.	Aug.	Sep.	Oct.	Jun.	Jul.	Aug.	Sep.	Oct	Jun.	Jul.	Aug.	Sep.	Oct
2016 (Shandong)	26.3	28.2	26.9	23.6	16.3	57.5	240.8	193.8	44.9	81.4	278.5	220.8	199.2	211.0	124.7
2016 (Sichuan)	24.4	25.8	26.4	21.4	17.8	47.4	270.7	56.1	121.9	27.8	133.0	143.2	175.5	61.5	43.8
2017 (Shandong)	26.3	28.6	27.5	23.3	15.0	63.7	203.7	59.6	35.2	38.4	236.6	229.8	219.4	172.5	118.4
2017 (Sichuan)	23.2	26.6	25.7	22.2	16.7	82	226.3	372.8	55.6	100.2	96.5	226.7	121.0	60.3	27.7

Supplemental Table S2. Average dark, light and dark-light (D:L) ratio in both locations (Shandong and Sichuan) during soybean growing seasons from 2016 to 2017.

Year	Dark (h)					Light (h)					Dark-Light ratio				
	Jun.	Jul.	Aug.	Sep.	Oct.	Jun.	Jul.	Aug.	Sep.	Oct	Jun.	Jul.	Aug.	Sep.	Oct
2016 (Shandong)	8.52	8.78	9.67	10.77	11.87	15.48	15.22	14.33	13.23	12.13	0.55	0.58	0.67	0.81	0.98
2016 (Sichuan)	8.98	9.20	9.93	10.87	11.78	15.02	14.80	14.07	13.13	12.22	0.60	0.62	0.71	0.83	0.96
2017 (Shandong)	8.52	8.78	9.65	10.77	11.85	15.48	15.22	14.35	13.23	12.15	0.55	0.58	0.67	0.81	0.98
2017 (Sichuan)	8.98	9.20	9.93	10.85	11.77	15.02	14.80	14.07	13.15	12.23	0.60	0.62	0.71	0.83	0.96

Supplemental Table S3. Primers sequence used in this study.

**Gene**	**Forward primer**	**Reverse primer**
GmTubulin	AACCTCCTCCTCATCGTACT	GACAGCATCAGCCATGTTCA
GmABA2	CATAGTCAACAATGCTGGAATCTC	ACCTAAGGCACTTGCTACAC
GmCYP707A1	GAGCAGATAGCGGATAATGT	GCACTCTTGTTCCTCAGTAA
GmRD29A	GGAAGGAAGAGCCAGTGA	AACCAAGAGCCAACAACAC
GmABI4	GAATCAACAGCAACAGCAACA	ACCGAAGAAGCATCCATAGC
GmABI5	CGAGTTCCAGCACAGTCT	TGTTCTCTTCAGCGTTCCA
GmGA3	AGATTGAACGCACCACACCTT	TCGCAGGAAGAAGAAGAGGATAGA
GmKAO	TTGGAGGAGGAAGTAGATTGTG	TGCTCTTAGGTTGTTGTAGATGAA
GmGA3ox1	GCCTCCTCCAAGACATTCAA	AGCCATCAACACCGTCAG
GmRGL1	TCCTCGGTCCATCTTCTCT	TTGTTGTTGTTGTTGTTGTTGTC
GmFT2a	GGATTGCCAGTTGCTGCTGT	GAGTGTGGGAGATTGCCAAT
