# Supplementary material for: Shading of the mother plant during seed development promotes subsequent seed germination in soybean
Source: J Exp Bot. 2020 Jan 11;71(6):2072–84. doi: 10.1093/jxb/erz553 (PMC7242070; doi:10.1093/jxb/erz553)
Supplement: erz553_suppl_supplementary_figures_S1_S6 [file erz553_suppl_supplementary_figures_s1_s6.pdf]

Chen et al., Fig. S1

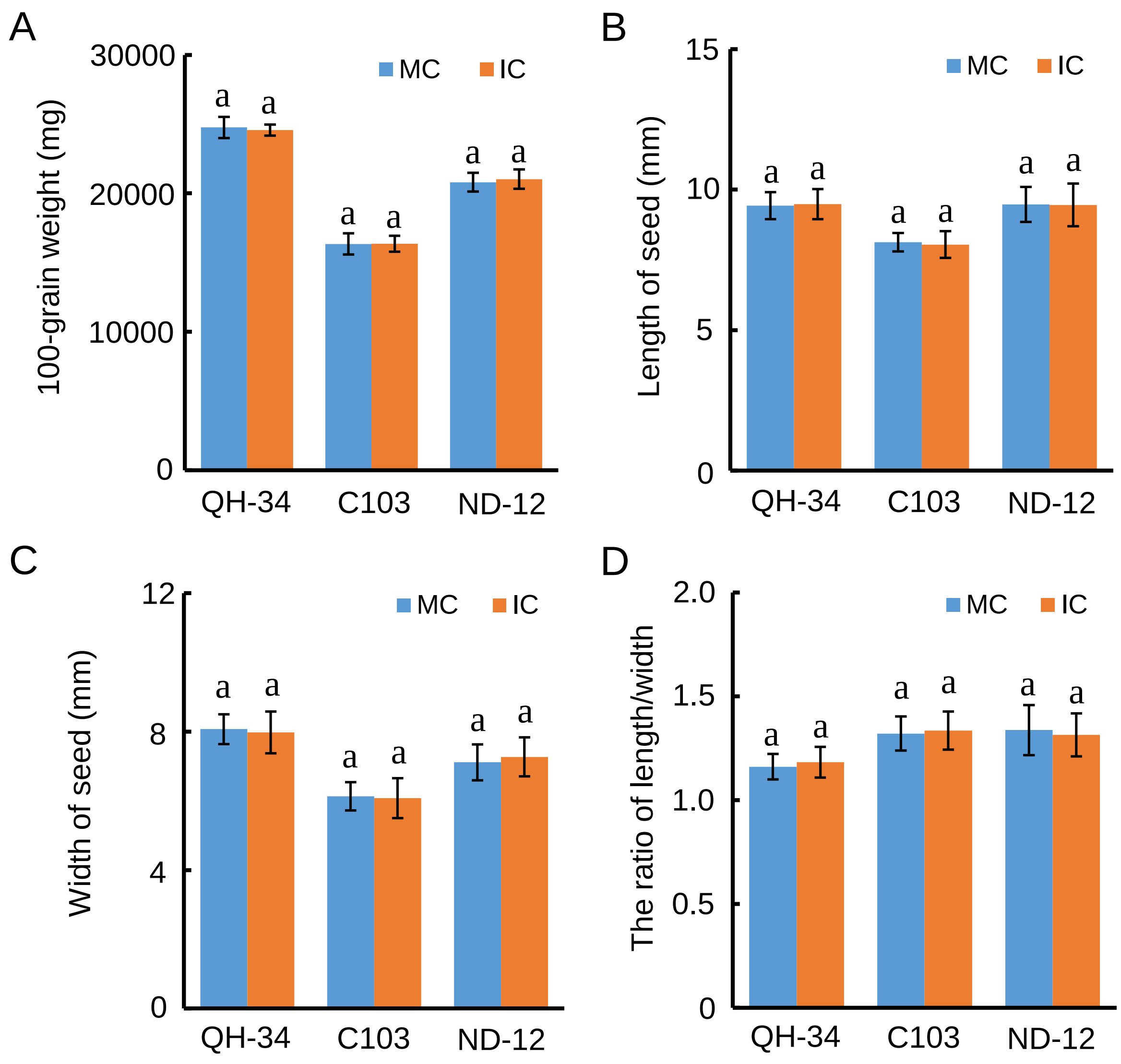

Supplemental Figure S1. Agronomic traits analysis of MC and IC soybean seeds.

The agronomic traits including 100-grain weight (A), length of seed (B), width of seed (C), and the ratio of seed length and width (D) were analyzed after harvest.

Chen et al., Fig. S2

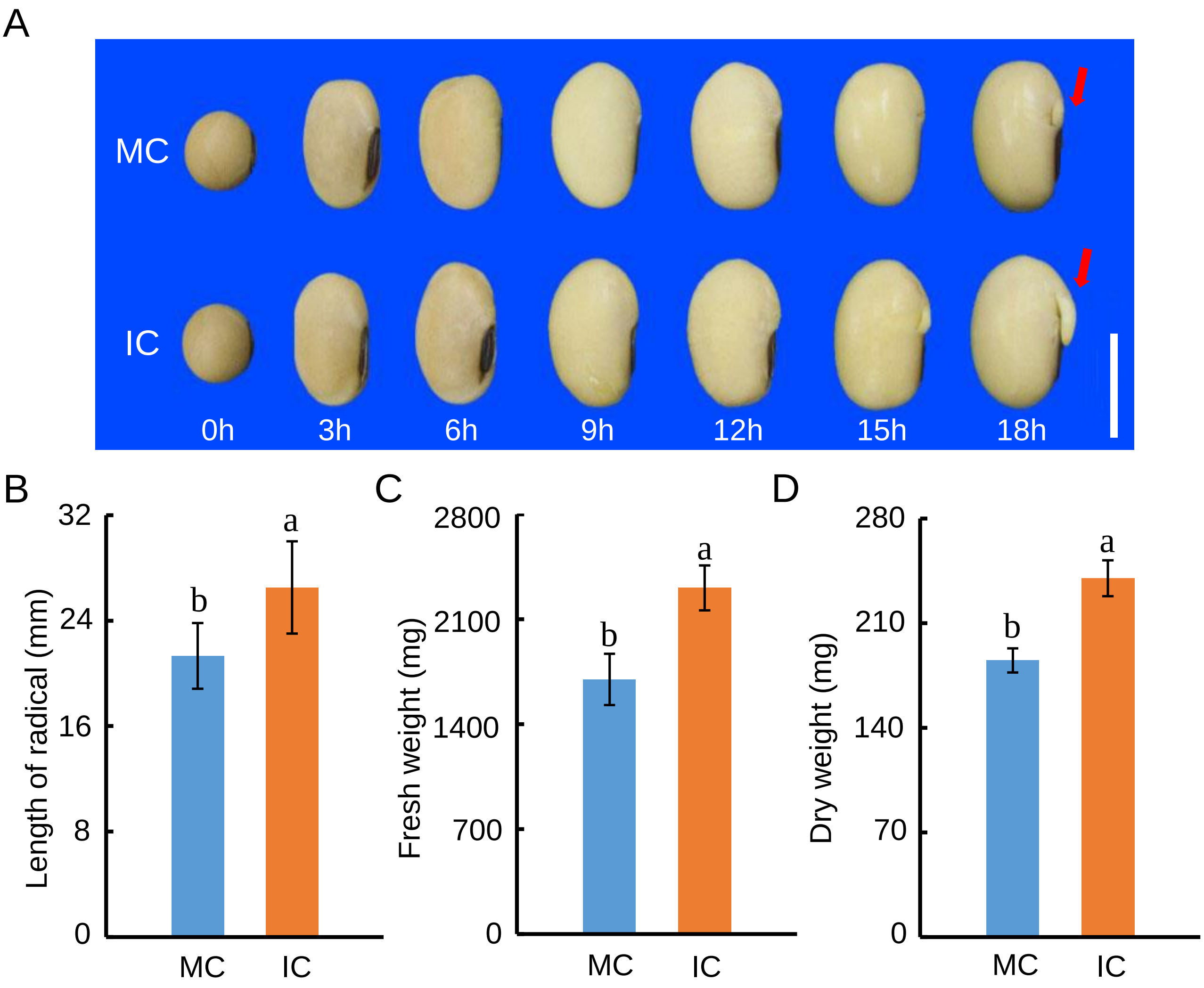

Supplemental Figure S2. Microscope observation of MC and IC seeds during imbibition, used cultivar QH-34.
Soybean QH-34 seeds (MC and IC) produced in Shandong location were employed for this experiment. The soybean seeds during imbibition were investigated by stereomicroscope (Leica M165 C, made in Germany) at different time points (0, 3, 6, 9, 12, 15 and 18 hours after sowing). **(A)** The representative photographs of seed coat rupture of MC and IC seeds during imbibition. **(B)** Radicle length of germinated soybean seeds at 48 hours after sowing. **(C)** and **(D)** Fresh and dry weight of root of germinated soybean seeds at 48 hours after sowing, respectively. Bar in panel A = 10 mm. The average percentages of four repeats ± SE are shown. Different letters indicate a significant difference at $P<0.05$ by Duncan's test analysis. MC: Monocropping; IC: Intercropping.

Chen et al., Fig. S3

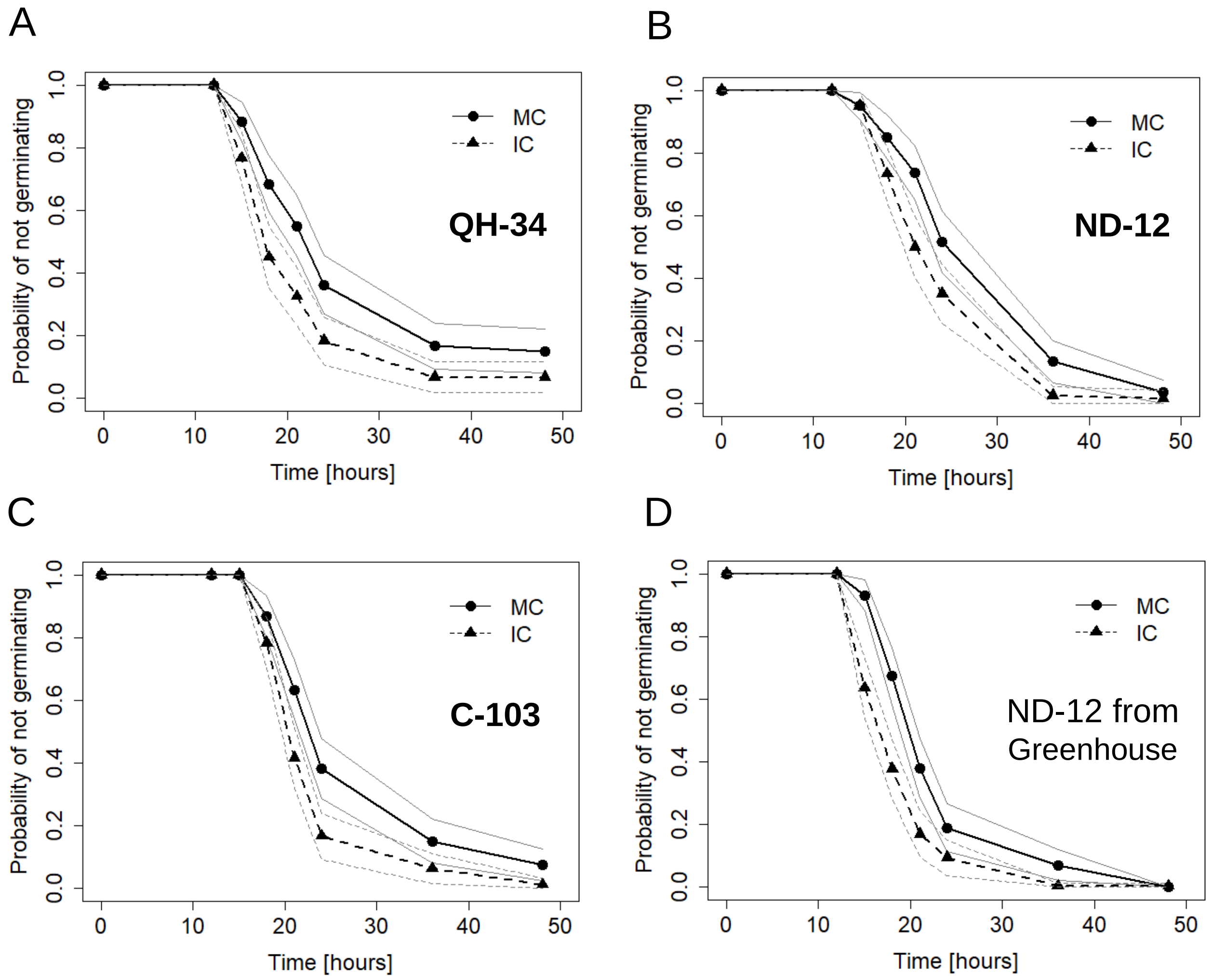

Supplemental Figure S3. Seed germination phenotype was confirmed by the life-table estimates.

The life-table estimates of survivor function for soybean seeds collected from IC (Shaded) and MC (CK) soybean seeds, computed by R function lifetab () from the KMsurv package. Three different soybean QH-34 seeds, produced in Shandong location (A), ND-12, produced in Sichuan location (B), and C-103, produced in Sichuan location (C) with monocropping and intercropping patterns were used to analyze. (D) ND-12 seeds produced in greenhouse under the shaded and right (CK) conditions were used to analyze. Point-wise 95% confidence intervals are based on a normal approximation using Greenwood standard errors computed by lifetab ().

Chen et al., Fig. S4

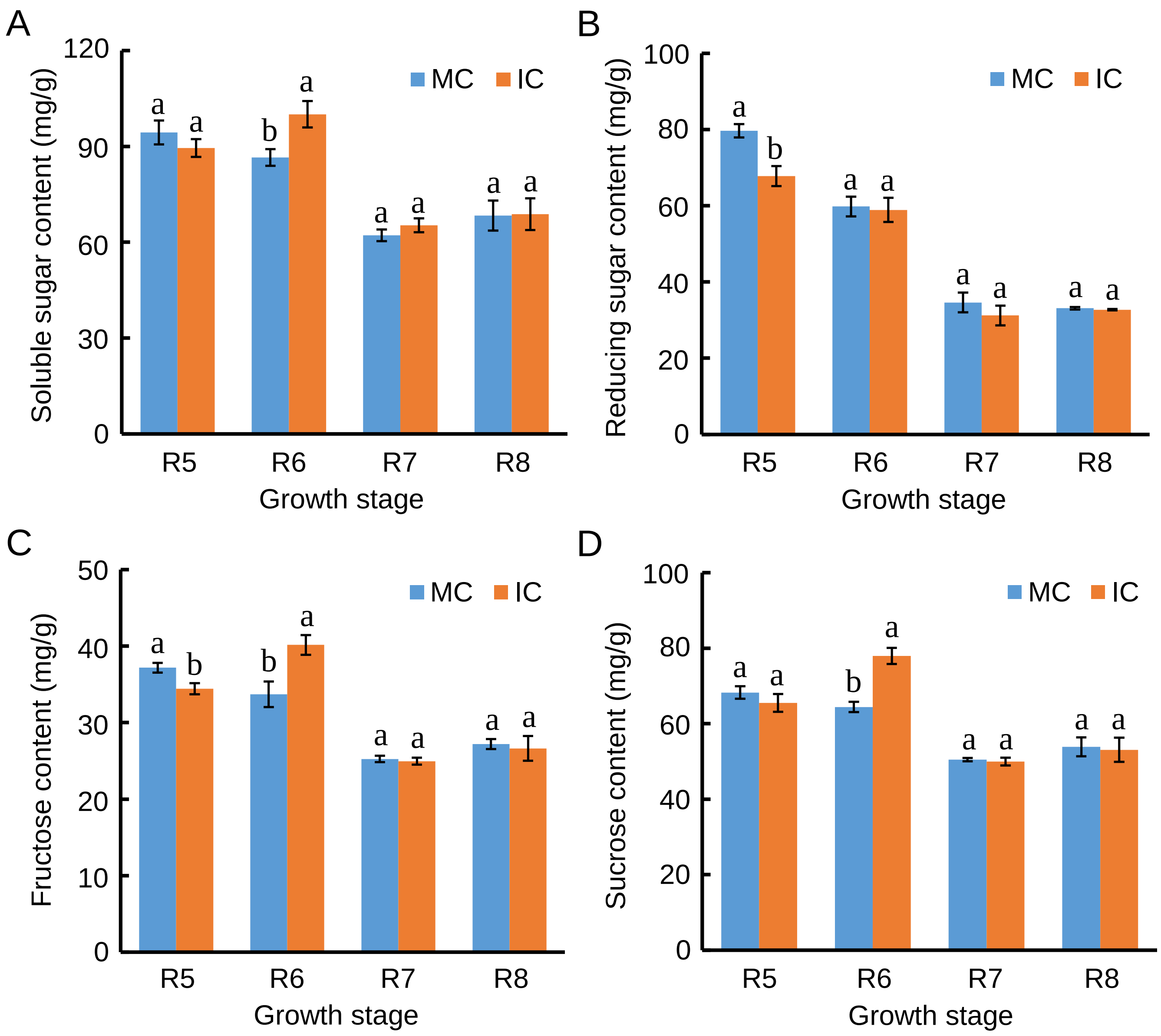

Supplemental Figure S4. Weak effect of shade on various sugars levels during soybean seed development.
Different types of samples, including R5, R6, R7 and R8 stages in MC and IC soybean seed development were sampled. **(A)** Soluble sugar quantification analysis. **(B)** Reducing sugar quantification. **(C)** Fructose concentration quantification. **(D)** Sucrose concentration analysis. The average percentages of four repeats ± SE are shown. Different letters indicate a significant difference at $P<0.05$ by Duncan's test analysis. MC: Monocropping; IC: Intercropping. Soybean ND12 seeds produced in Sichuan location was employed for this experiment.

Chen et al., Fig. S5

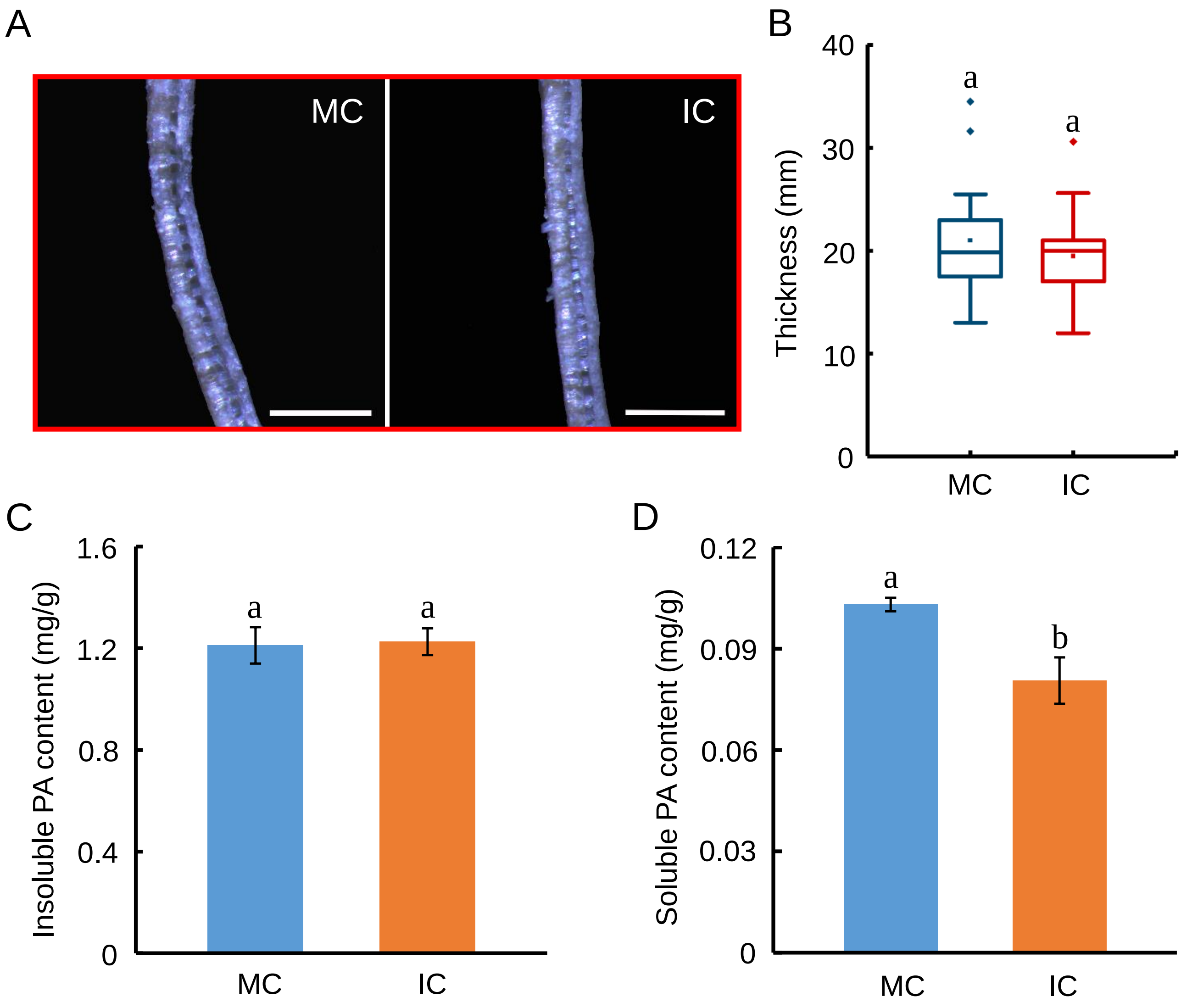

Supplemental Figure S5. Thickness and PAs quantification analysis in seed coat, used soybean cultivar QH-34.
Soybean QH-34 seeds (MC and IC) produced in Shandong location were employed for this experiment. **(A)** Representative images of MC and IC soybean seed coat. **(B)** Quantitative analysis of MC and IC seed coat thickness. **(C-D)** Insoluble and soluble PAs quantification in MC and IC seed coat. MC and IC seed coats were investigated by stereomicroscope (Leica M165 C, made in Germany). At least ten seeds were used to measure the thickness of seed coat, and each seed was measured five times with different angles. Bar=50 μm. The average percentages of four repeats ± SE are shown. Different letters indicate a significant difference at $P<0.05$ by Duncan's test analysis. MC: Monocropping; IC: Intercropping.

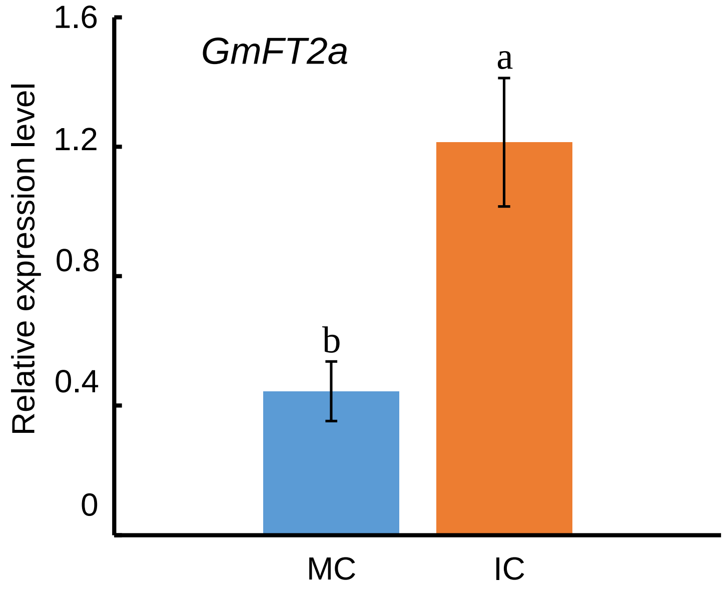

Supplemental Figure S6. *GmFT2a* gene expression analysis in MC and IC soybean seeds.
Different letters indicate a significant difference at $P<0.05$ by Duncan's test analysis. MC: Monocropping; IC: Intercropping. Soybean ND-12 seeds produced in Sichuan location were employed for this experiment.
